# *N*-(2,4-Dichloro­phen­yl)benzene­sulfonamide

**DOI:** 10.1107/S160053680905452X

**Published:** 2009-12-24

**Authors:** B. Thimme Gowda, Sabine Foro, P. G. Nirmala, Hartmut Fuess

**Affiliations:** a Department of Chemistry, Mangalore University, Mangalagangotri 574 199, Mangalore, India; b Institute of Materials Science, Darmstadt University of Technology, Petersenstrasse 23, D-64287 Darmstadt, Germany

## Abstract

The title compound, C_12_H_9_Cl_2_NO_2_S, crystallizes with two independent mol­ecules in the asymmetric unit. The dihedral angles between the two aromatic rings are 70.8 (1) and 74.8 (1)° for the two mol­ecules. The crystal structure features dimers made up of one each of the two asymmetric molecules linked by pairs of N—H⋯O hydrogen bonds. An intra­molecular N—H⋯Cl hydrogen bond is also observed in both mol­ecules.

## Related literature

For the preparation of the title compound, see: Shetty & Gowda (2005 ▶). For our study of the effect of substituents on the structures of *N*-(ar­yl)aryl­sulfonamides, see: Gowda *et al.* (2008 ▶; 2010 ▶). For related structures, see: Gelbrich *et al.* (2007 ▶); Perlovich *et al.* (2006 ▶).
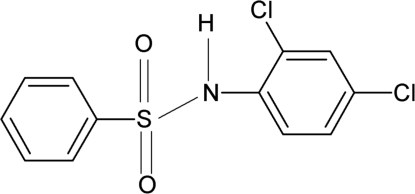


## Experimental

### 

#### Crystal data

C_12_H_9_Cl_2_NO_2_S
*M* *_r_* = 302.16
Monoclinic, 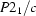

*a* = 8.2428 (6) Å
*b* = 19.473 (1) Å
*c* = 16.873 (1) Å
β = 103.317 (7)°
*V* = 2635.5 (3) Å^3^
*Z* = 8
Mo *K*α radiation
μ = 0.64 mm^−1^
*T* = 299 K
0.50 × 0.24 × 0.14 mm

#### Data collection

Oxford Diffraction Xcalibur diffractometer with a Sapphire CCD detector
Absorption correction: multi-scan (*CrysAlis RED*; Oxford Diffraction, 2009 ▶) *T*_min_ = 0.740, *T*_max_ = 0.915
17396 measured reflections
4807 independent reflections
3444 reflections with *I* > 2σ(*I*)
*R*_int_ = 0.029

#### Refinement

*R*[*F* ^2^ > 2σ(*F* ^2^)] = 0.048
*wR*(*F* ^2^) = 0.106
*S* = 1.08
4807 reflections
331 parameters
2 restraints
H atoms treated by a mixture of independent and constrained refinement
Δρ_max_ = 0.29 e Å^−3^
Δρ_min_ = −0.27 e Å^−3^

### 

Data collection: *CrysAlis CCD* (Oxford Diffraction, 2009 ▶); cell refinement: *CrysAlis RED* (Oxford Diffraction, 2009 ▶); data reduction: *CrysAlis RED*; program(s) used to solve structure: *SHELXS97* (Sheldrick, 2008 ▶); program(s) used to refine structure: *SHELXL97* (Sheldrick, 2008 ▶); molecular graphics: *PLATON* (Spek, 2009 ▶); software used to prepare material for publication: *SHELXL97* ▶.

## Supplementary Material

Crystal structure: contains datablocks I, global. DOI: 10.1107/S160053680905452X/bt5148sup1.cif

Structure factors: contains datablocks I. DOI: 10.1107/S160053680905452X/bt5148Isup2.hkl

Additional supplementary materials:  crystallographic information; 3D view; checkCIF report

## Figures and Tables

**Table 1:** Hydrogen-bond geometry (Å, °)

*D*—H⋯*A*	*D*—H	H⋯*A*	*D*⋯*A*	*D*—H⋯*A*
N1—H1*N*⋯O4	0.85 (1)	2.19 (1)	3.015 (3)	165 (3)
N1—H1*N*⋯Cl1	0.85 (1)	2.60 (3)	2.998 (3)	110 (2)
N2—H2*N*⋯O2	0.85 (1)	2.38 (2)	3.192 (4)	160 (3)
N2—H2*N*⋯Cl3	0.85 (1)	2.55 (3)	2.996 (3)	113 (3)

**Fig. 1.**
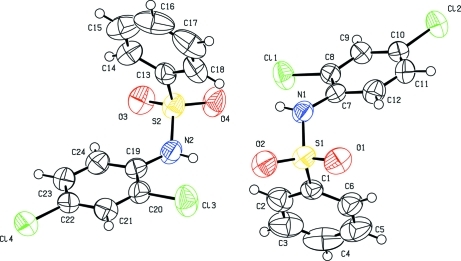
Molecular structure of (I), showing the atom labelling and displacement ellipsoids drawn at the 50% probability level.

**Fig. 2.**
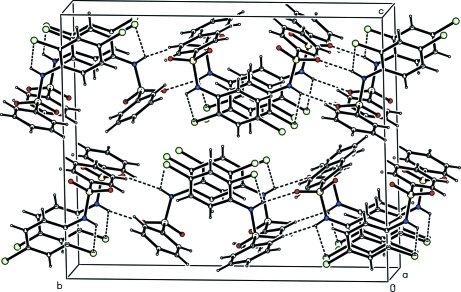
Molecular packing of (I) with hydrogen bonding shown as dashed lines.

## supplementary crystallographic information

### Comment

In the present work, as part of a study of substituent effects on the structures of *N*-(aryl)arylsulfonamides (Gowda *et al.*, 2008; Gowda *et al.*, 2010), the structure of *N*-(2,4-Dichlorophenyl)benzenesulfonamide (I) has been determined. The asymmetric unit of the structure contains two independent molecules.

The sulfonyl benzene and the aniline benzene rings in the two molecules of (I) are tilted relative to each other by 70.8 (1)° (molecule 1) and 74.8 (1)° (molecule 2). The other bond parameters in (I) are similar to those observed in other aryl sulfonamides (Gowda *et al.*, 2010; Perlovich *et al.*, 2006; Gelbrich *et al.*, 2007).

In the crystal structure, both the intramolecular N—H···Cl and intermolecular N—H···O hydrogen bonds are observed. The pairs of intermolecular N–H···O hydrogen bonds (Table 1) link the molecules through inversion-related dimers (Fig. 2).

### Experimental

The solution of benzene (10 ml) in chloroform (40 ml) was treated dropwise with chlorosulfonic acid (25 ml) at 0 ° C. After the initial evolution of hydrogen chloride subsided, the reaction mixture was brought to room temperature and poured into crushed ice in a beaker. The chloroform layer was separated, washed with cold water and allowed to evaporate slowly. The residual benzenesulfonylchloride was treated with 2,4-dichloroaniline in the stoichiometric amounts and boiled for ten minutes. The reaction mixture was then cooled to room temperature and added to ice cold water (100 ml). The resultant solid *N*-(2,4-dichlorophenyl)benzenesulfonamide was filtered under suction and washed thoroughly with cold water. It was then recrystallized to constant melting point from dilute ethanol. The purity of the compound was checked and characterized by recording its infrared and NMR spectra (Shetty & Gowda, 2005). The rod like colorless single crystals used in X-ray diffraction studies were grown in ethanolic solution by evaporating it at room temperature.

### Refinement

The H atoms of the NH groups were located in a difference map and refined with a N-H distance restraint of 0.86 (1) Å. The other H atoms were positioned with idealized geometry using a riding model with C—H = 0.93 Å A l l H atoms were refined with isotropic displacement parameters (set to 1.2 times of the *U*_eq_ of the parent atom).

### Crystal data

**Table:**

C_12_H_9_Cl_2_NO_2_S	*F*(000) = 1232
*M**_r_* = 302.16	*D*_x_ = 1.523 Mg m^−^^3^
Monoclinic, *P*2_1_/*c*	Mo *K*α radiation, λ = 0.71073 Å
Hall symbol: -P 2ybc	Cell parameters from 6373 reflections
*a* = 8.2428 (6) Å	θ = 2.4–28.2°
*b* = 19.473 (1) Å	µ = 0.64 mm^−^^1^
*c* = 16.873 (1) Å	*T* = 299 K
β = 103.317 (7)°	Rod, colourless
*V* = 2635.5 (3) Å^3^	0.50 × 0.24 × 0.14 mm
*Z* = 8	

### Data collection

**Table:**

Oxford Diffraction Xcalibur diffractometer with a Sapphire CCD detector	4807 independent reflections
Radiation source: fine-focus sealed tube	3444 reflections with *I* > 2σ(*I*)
graphite	*R*_int_ = 0.029
Rotation method data acquisition using ω and phi scans	θ_max_ = 25.4°, θ_min_ = 2.4°
Absorption correction: multi-scan (*CrysAlis RED*; Oxford Diffraction, 2009)	*h* = −9→8
*T*_min_ = 0.740, *T*_max_ = 0.915	*k* = −23→20
17396 measured reflections	*l* = −19→20

### Refinement

**Table:**

Refinement on *F*^2^	Primary atom site location: structure-invariant direct methods
Least-squares matrix: full	Secondary atom site location: difference Fourier map
*R*[*F*^2^ > 2σ(*F*^2^)] = 0.048	Hydrogen site location: inferred from neighbouring sites
*wR*(*F*^2^) = 0.106	H atoms treated by a mixture of independent and constrained refinement
*S* = 1.08	*w* = 1/[σ^2^(*F*_o_^2^) + (0.0259*P*)^2^ + 2.4186*P*] where *P* = (*F*_o_^2^ + 2*F*_c_^2^)/3
4807 reflections	(Δ/σ)_max_ = 0.003
331 parameters	Δρ_max_ = 0.28 e Å^−^^3^
2 restraints	Δρ_min_ = −0.27 e Å^−^^3^

### Special details

**Table:**

Experimental. CrysAlis RED (Oxford Diffraction, 2009) Empirical absorption correction using spherical harmonics, implemented in SCALE3 ABSPACK scaling algorithm.
Geometry. All e.s.d.'s (except the e.s.d. in the dihedral angle between two l.s. planes) are estimated using the full covariance matrix. The cell e.s.d.'s are taken into account individually in the estimation of e.s.d.'s in distances, angles and torsion angles; correlations between e.s.d.'s in cell parameters are only used when they are defined by crystal symmetry. An approximate (isotropic) treatment of cell e.s.d.'s is used for estimating e.s.d.'s involving l.s. planes.
Refinement. Refinement of *F*^2^ against ALL reflections. The weighted *R*-factor *wR* and goodness of fit *S* are based on *F*^2^, conventional *R*-factors *R* are based on *F*, with *F* set to zero for negative *F*^2^. The threshold expression of *F*^2^ > σ(*F*^2^) is used only for calculating *R*-factors(gt) *etc*. and is not relevant to the choice of reflections for refinement. *R*-factors based on *F*^2^ are statistically about twice as large as those based on *F*, and *R*- factors based on ALL data will be even larger.

### Fractional atomic coordinates and isotropic or equivalent isotropic displacement parameters (Å^2^)

**Table:**

	*x*	*y*	*z*	*U*_iso_*/*U*_eq_	
Cl1	0.07684 (12)	0.41223 (4)	0.39801 (6)	0.0729 (3)	
Cl2	0.12156 (15)	0.68538 (5)	0.42889 (7)	0.0901 (3)	
S1	0.18167 (10)	0.41573 (4)	0.15935 (5)	0.0567 (2)	
O1	0.2392 (3)	0.47082 (13)	0.11774 (14)	0.0746 (7)	
O2	0.2379 (3)	0.34755 (12)	0.14983 (14)	0.0769 (7)	
N1	0.2373 (3)	0.43048 (13)	0.25698 (15)	0.0548 (6)	
H1N	0.235 (4)	0.3935 (10)	0.2832 (17)	0.066*	
C1	−0.0361 (4)	0.41698 (16)	0.13336 (17)	0.0509 (7)	
C2	−0.1220 (5)	0.3600 (2)	0.1486 (2)	0.0739 (10)	
H2	−0.0650	0.3212	0.1722	0.089*	
C3	−0.2925 (6)	0.3610 (3)	0.1289 (3)	0.0982 (14)	
H3	−0.3518	0.3225	0.1387	0.118*	
C4	−0.3756 (5)	0.4182 (4)	0.0949 (3)	0.1036 (17)	
H4	−0.4915	0.4185	0.0822	0.124*	
C5	−0.2897 (6)	0.4757 (3)	0.0791 (2)	0.0952 (14)	
H5	−0.3476	0.5144	0.0557	0.114*	
C6	−0.1185 (5)	0.47538 (19)	0.0982 (2)	0.0706 (10)	
H6	−0.0591	0.5136	0.0878	0.085*	
C7	0.2003 (3)	0.49219 (15)	0.29451 (17)	0.0478 (7)	
C8	0.1319 (4)	0.49032 (15)	0.36246 (18)	0.0490 (7)	
C9	0.1073 (4)	0.54913 (16)	0.40335 (19)	0.0572 (8)	
H9	0.0632	0.5467	0.4493	0.069*	
C10	0.1487 (4)	0.61158 (16)	0.3756 (2)	0.0595 (8)	
C11	0.2117 (5)	0.61546 (17)	0.3073 (2)	0.0692 (10)	
H11	0.2359	0.6580	0.2878	0.083*	
C12	0.2390 (4)	0.55639 (17)	0.2676 (2)	0.0655 (9)	
H12	0.2842	0.5594	0.2220	0.079*	
Cl3	0.36502 (15)	0.20468 (5)	0.06873 (6)	0.0908 (3)	
Cl4	0.40560 (13)	−0.07047 (5)	0.08844 (6)	0.0814 (3)	
S2	0.31006 (11)	0.21769 (4)	0.32166 (5)	0.0617 (2)	
O3	0.2519 (3)	0.16658 (13)	0.36835 (14)	0.0768 (7)	
O4	0.2532 (3)	0.28666 (12)	0.32451 (15)	0.0851 (8)	
N2	0.2533 (4)	0.19672 (14)	0.22530 (17)	0.0633 (7)	
H2N	0.261 (4)	0.2319 (11)	0.1965 (18)	0.076*	
C13	0.5279 (4)	0.21634 (15)	0.34783 (18)	0.0530 (8)	
C14	0.6093 (5)	0.1640 (2)	0.3963 (2)	0.0707 (10)	
H14	0.5492	0.1293	0.4145	0.085*	
C15	0.7802 (6)	0.1639 (3)	0.4173 (3)	0.1001 (15)	
H15	0.8372	0.1289	0.4497	0.120*	
C16	0.8680 (6)	0.2161 (4)	0.3898 (3)	0.1076 (18)	
H16	0.9839	0.2168	0.4048	0.129*	
C17	0.7841 (7)	0.2664 (3)	0.3409 (3)	0.1026 (15)	
H17	0.8437	0.3007	0.3217	0.123*	
C18	0.6148 (5)	0.26737 (18)	0.3197 (2)	0.0775 (11)	
H18	0.5587	0.3021	0.2865	0.093*	
C19	0.2931 (4)	0.13263 (16)	0.19385 (19)	0.0546 (8)	
C20	0.3432 (4)	0.12956 (16)	0.1208 (2)	0.0595 (8)	
C21	0.3763 (4)	0.06739 (17)	0.0877 (2)	0.0638 (9)	
H21	0.4081	0.0661	0.0383	0.077*	
C22	0.3616 (4)	0.00774 (16)	0.1289 (2)	0.0610 (8)	
C23	0.3133 (4)	0.00912 (18)	0.2012 (2)	0.0689 (9)	
H23	0.3051	−0.0315	0.2289	0.083*	
C24	0.2771 (4)	0.07091 (18)	0.2327 (2)	0.0678 (9)	
H24	0.2412	0.0714	0.2811	0.081*	

### Atomic displacement parameters (Å^2^)

**Table:**

	*U*^11^	*U*^22^	*U*^33^	*U*^12^	*U*^13^	*U*^23^
Cl1	0.0987 (7)	0.0494 (5)	0.0798 (6)	−0.0115 (4)	0.0397 (5)	−0.0012 (4)
Cl2	0.1147 (8)	0.0507 (5)	0.0958 (7)	0.0072 (5)	0.0053 (6)	−0.0148 (5)
S1	0.0551 (5)	0.0656 (5)	0.0520 (5)	0.0121 (4)	0.0177 (4)	0.0008 (4)
O1	0.0718 (16)	0.0927 (18)	0.0660 (15)	−0.0021 (13)	0.0298 (13)	0.0118 (13)
O2	0.0847 (17)	0.0774 (16)	0.0708 (15)	0.0323 (13)	0.0222 (13)	−0.0079 (13)
N1	0.0551 (16)	0.0541 (16)	0.0532 (16)	0.0088 (13)	0.0082 (13)	0.0053 (12)
C1	0.0545 (19)	0.0586 (19)	0.0400 (16)	0.0047 (15)	0.0118 (14)	−0.0060 (14)
C2	0.073 (3)	0.077 (3)	0.072 (2)	−0.010 (2)	0.016 (2)	−0.0035 (19)
C3	0.079 (3)	0.135 (4)	0.080 (3)	−0.033 (3)	0.017 (2)	−0.005 (3)
C4	0.051 (3)	0.192 (6)	0.068 (3)	0.002 (3)	0.015 (2)	−0.030 (3)
C5	0.076 (3)	0.127 (4)	0.075 (3)	0.040 (3)	0.002 (2)	−0.010 (3)
C6	0.070 (3)	0.075 (2)	0.063 (2)	0.0159 (19)	0.0080 (18)	0.0001 (18)
C7	0.0406 (16)	0.0490 (17)	0.0489 (17)	0.0036 (13)	0.0005 (13)	0.0017 (14)
C8	0.0453 (17)	0.0469 (17)	0.0531 (18)	−0.0034 (13)	0.0074 (14)	0.0021 (14)
C9	0.059 (2)	0.0536 (19)	0.0576 (19)	−0.0005 (15)	0.0108 (16)	−0.0031 (15)
C10	0.060 (2)	0.0493 (18)	0.061 (2)	0.0019 (15)	−0.0032 (16)	−0.0017 (15)
C11	0.083 (3)	0.0471 (19)	0.071 (2)	−0.0058 (17)	0.005 (2)	0.0095 (17)
C12	0.074 (2)	0.064 (2)	0.058 (2)	−0.0045 (17)	0.0152 (18)	0.0104 (17)
Cl3	0.1349 (10)	0.0616 (6)	0.0874 (7)	−0.0052 (6)	0.0490 (7)	0.0099 (5)
Cl4	0.0930 (7)	0.0606 (5)	0.0820 (6)	0.0015 (5)	0.0025 (5)	−0.0108 (5)
S2	0.0679 (6)	0.0615 (5)	0.0598 (5)	0.0156 (4)	0.0229 (4)	0.0039 (4)
O3	0.0781 (17)	0.0868 (17)	0.0751 (16)	0.0024 (13)	0.0372 (13)	0.0106 (13)
O4	0.110 (2)	0.0681 (16)	0.0819 (17)	0.0393 (15)	0.0314 (15)	0.0032 (13)
N2	0.0685 (19)	0.0600 (18)	0.0595 (17)	0.0116 (15)	0.0109 (14)	0.0104 (14)
C13	0.068 (2)	0.0483 (17)	0.0463 (17)	0.0016 (15)	0.0202 (15)	−0.0071 (14)
C14	0.068 (3)	0.084 (3)	0.061 (2)	0.010 (2)	0.0156 (18)	0.0069 (19)
C15	0.076 (3)	0.145 (4)	0.073 (3)	0.030 (3)	0.005 (2)	−0.009 (3)
C16	0.058 (3)	0.175 (6)	0.090 (3)	−0.012 (3)	0.017 (3)	−0.057 (4)
C17	0.099 (4)	0.107 (4)	0.110 (4)	−0.045 (3)	0.040 (3)	−0.039 (3)
C18	0.092 (3)	0.059 (2)	0.086 (3)	−0.015 (2)	0.029 (2)	−0.0129 (19)
C19	0.0486 (19)	0.0563 (19)	0.0548 (19)	0.0012 (15)	0.0034 (15)	0.0011 (15)
C20	0.059 (2)	0.056 (2)	0.061 (2)	−0.0044 (16)	0.0083 (16)	0.0056 (16)
C21	0.065 (2)	0.065 (2)	0.061 (2)	−0.0037 (17)	0.0136 (17)	−0.0050 (17)
C22	0.058 (2)	0.054 (2)	0.063 (2)	−0.0016 (16)	−0.0013 (17)	−0.0025 (16)
C23	0.081 (3)	0.057 (2)	0.064 (2)	−0.0058 (18)	0.0058 (19)	0.0079 (17)
C24	0.076 (2)	0.069 (2)	0.059 (2)	−0.0018 (18)	0.0139 (18)	0.0061 (18)

### Geometric parameters (Å, °)

**Table:**

Cl1—C8	1.733 (3)	Cl3—C20	1.737 (3)
Cl2—C10	1.737 (3)	Cl4—C22	1.741 (3)
S1—O1	1.422 (2)	S2—O3	1.419 (2)
S1—O2	1.428 (2)	S2—O4	1.427 (2)
S1—N1	1.631 (3)	S2—N2	1.637 (3)
S1—C1	1.747 (3)	S2—C13	1.747 (3)
N1—C7	1.424 (4)	N2—C19	1.424 (4)
N1—H1N	0.847 (10)	N2—H2N	0.851 (10)
C1—C2	1.372 (4)	C13—C18	1.373 (4)
C1—C6	1.386 (4)	C13—C14	1.381 (4)
C2—C3	1.367 (5)	C14—C15	1.371 (5)
C2—H2	0.9300	C14—H14	0.9300
C3—C4	1.362 (6)	C15—C16	1.388 (7)
C3—H3	0.9300	C15—H15	0.9300
C4—C5	1.384 (7)	C16—C17	1.363 (7)
C4—H4	0.9300	C16—H16	0.9300
C5—C6	1.373 (5)	C17—C18	1.358 (6)
C5—H5	0.9300	C17—H17	0.9300
C6—H6	0.9300	C18—H18	0.9300
C7—C8	1.390 (4)	C19—C20	1.389 (4)
C7—C12	1.392 (4)	C19—C24	1.390 (4)
C8—C9	1.376 (4)	C20—C21	1.386 (4)
C9—C10	1.374 (4)	C21—C22	1.373 (4)
C9—H9	0.9300	C21—H21	0.9300
C10—C11	1.370 (5)	C22—C23	1.368 (5)
C11—C12	1.376 (5)	C23—C24	1.376 (5)
C11—H11	0.9300	C23—H23	0.9300
C12—H12	0.9300	C24—H24	0.9300
			
O1—S1—O2	119.49 (15)	O3—S2—O4	119.04 (16)
O1—S1—N1	108.53 (15)	O3—S2—N2	108.69 (15)
O2—S1—N1	104.74 (14)	O4—S2—N2	104.32 (15)
O1—S1—C1	107.80 (15)	O3—S2—C13	107.94 (15)
O2—S1—C1	109.05 (15)	O4—S2—C13	109.40 (16)
N1—S1—C1	106.55 (14)	N2—S2—C13	106.83 (14)
C7—N1—S1	124.0 (2)	C19—N2—S2	123.4 (2)
C7—N1—H1N	117 (2)	C19—N2—H2N	116 (2)
S1—N1—H1N	110 (2)	S2—N2—H2N	109 (2)
C2—C1—C6	121.3 (3)	C18—C13—C14	121.2 (3)
C2—C1—S1	119.1 (3)	C18—C13—S2	119.3 (3)
C6—C1—S1	119.5 (3)	C14—C13—S2	119.4 (3)
C3—C2—C1	119.3 (4)	C15—C14—C13	118.9 (4)
C3—C2—H2	120.4	C15—C14—H14	120.5
C1—C2—H2	120.4	C13—C14—H14	120.5
C4—C3—C2	120.2 (4)	C14—C15—C16	119.8 (5)
C4—C3—H3	119.9	C14—C15—H15	120.1
C2—C3—H3	119.9	C16—C15—H15	120.1
C3—C4—C5	120.8 (4)	C17—C16—C15	119.9 (4)
C3—C4—H4	119.6	C17—C16—H16	120.1
C5—C4—H4	119.6	C15—C16—H16	120.1
C6—C5—C4	119.7 (4)	C18—C17—C16	121.1 (5)
C6—C5—H5	120.2	C18—C17—H17	119.4
C4—C5—H5	120.2	C16—C17—H17	119.4
C5—C6—C1	118.7 (4)	C17—C18—C13	119.1 (4)
C5—C6—H6	120.7	C17—C18—H18	120.5
C1—C6—H6	120.7	C13—C18—H18	120.5
C8—C7—C12	117.4 (3)	C20—C19—C24	117.5 (3)
C8—C7—N1	120.9 (3)	C20—C19—N2	120.7 (3)
C12—C7—N1	121.6 (3)	C24—C19—N2	121.8 (3)
C9—C8—C7	121.8 (3)	C21—C20—C19	121.4 (3)
C9—C8—Cl1	118.4 (2)	C21—C20—Cl3	118.6 (3)
C7—C8—Cl1	119.8 (2)	C19—C20—Cl3	119.9 (3)
C10—C9—C8	119.3 (3)	C22—C21—C20	119.1 (3)
C10—C9—H9	120.4	C22—C21—H21	120.4
C8—C9—H9	120.4	C20—C21—H21	120.4
C11—C10—C9	120.5 (3)	C23—C22—C21	120.8 (3)
C11—C10—Cl2	120.6 (3)	C23—C22—Cl4	119.8 (3)
C9—C10—Cl2	118.9 (3)	C21—C22—Cl4	119.4 (3)
C10—C11—C12	120.0 (3)	C22—C23—C24	119.7 (3)
C10—C11—H11	120.0	C22—C23—H23	120.2
C12—C11—H11	120.0	C24—C23—H23	120.2
C11—C12—C7	121.1 (3)	C23—C24—C19	121.4 (3)
C11—C12—H12	119.5	C23—C24—H24	119.3
C7—C12—H12	119.5	C19—C24—H24	119.3
			
O1—S1—N1—C7	53.7 (3)	O3—S2—N2—C19	−55.5 (3)
O2—S1—N1—C7	−177.6 (2)	O4—S2—N2—C19	176.5 (3)
C1—S1—N1—C7	−62.1 (3)	C13—S2—N2—C19	60.7 (3)
O1—S1—C1—C2	163.9 (2)	O3—S2—C13—C18	−170.9 (3)
O2—S1—C1—C2	32.8 (3)	O4—S2—C13—C18	−40.0 (3)
N1—S1—C1—C2	−79.8 (3)	N2—S2—C13—C18	72.4 (3)
O1—S1—C1—C6	−16.4 (3)	O3—S2—C13—C14	9.2 (3)
O2—S1—C1—C6	−147.6 (2)	O4—S2—C13—C14	140.1 (3)
N1—S1—C1—C6	99.9 (3)	N2—S2—C13—C14	−107.5 (3)
C6—C1—C2—C3	−0.1 (5)	C18—C13—C14—C15	0.9 (5)
S1—C1—C2—C3	179.5 (3)	S2—C13—C14—C15	−179.2 (3)
C1—C2—C3—C4	−0.4 (6)	C13—C14—C15—C16	0.2 (6)
C2—C3—C4—C5	0.6 (7)	C14—C15—C16—C17	−1.3 (7)
C3—C4—C5—C6	−0.3 (7)	C15—C16—C17—C18	1.5 (7)
C4—C5—C6—C1	−0.2 (6)	C16—C17—C18—C13	−0.4 (6)
C2—C1—C6—C5	0.4 (5)	C14—C13—C18—C17	−0.8 (5)
S1—C1—C6—C5	−179.2 (3)	S2—C13—C18—C17	179.3 (3)
S1—N1—C7—C8	131.2 (3)	S2—N2—C19—C20	−138.3 (3)
S1—N1—C7—C12	−52.3 (4)	S2—N2—C19—C24	44.4 (4)
C12—C7—C8—C9	−1.8 (4)	C24—C19—C20—C21	−0.1 (5)
N1—C7—C8—C9	174.9 (3)	N2—C19—C20—C21	−177.5 (3)
C12—C7—C8—Cl1	178.7 (2)	C24—C19—C20—Cl3	179.8 (2)
N1—C7—C8—Cl1	−4.6 (4)	N2—C19—C20—Cl3	2.3 (4)
C7—C8—C9—C10	1.2 (5)	C19—C20—C21—C22	−1.0 (5)
Cl1—C8—C9—C10	−179.4 (2)	Cl3—C20—C21—C22	179.2 (3)
C8—C9—C10—C11	0.9 (5)	C20—C21—C22—C23	0.6 (5)
C8—C9—C10—Cl2	−178.6 (2)	C20—C21—C22—Cl4	−179.2 (2)
C9—C10—C11—C12	−2.2 (5)	C21—C22—C23—C24	0.8 (5)
Cl2—C10—C11—C12	177.3 (3)	Cl4—C22—C23—C24	−179.4 (3)
C10—C11—C12—C7	1.5 (5)	C22—C23—C24—C19	−1.9 (5)
C8—C7—C12—C11	0.5 (5)	C20—C19—C24—C23	1.5 (5)
N1—C7—C12—C11	−176.2 (3)	N2—C19—C24—C23	178.9 (3)

### Hydrogen-bond geometry (Å, °)

**Table:**

*D*—H···*A*	*D*—H	H···*A*	*D*···*A*	*D*—H···*A*
N1—H1N···O4	0.85 (1)	2.19 (1)	3.015 (3)	165 (3)
N1—H1N···Cl1	0.85 (1)	2.60 (3)	2.998 (3)	110 (2)
N2—H2N···O2	0.85 (1)	2.38 (2)	3.192 (4)	160 (3)
N2—H2N···Cl3	0.85 (1)	2.55 (3)	2.996 (3)	113 (3)

## References

[bb1] Gelbrich, T., Hursthouse, M. B. & Threlfall, T. L. (2007). *Acta Cryst.* B**63**, 621–632. 10.1107/S010876810701395X 17641433

[bb2] Gowda, B. T., Foro, S., Babitha, K. S. & Fuess, H. (2008). *Acta Cryst.* E**64**, o2190. 10.1107/S1600536808034351 PMC2959669 21581048

[bb3] Gowda, B. T., Foro, S., Nirmala, P. G. & Fuess, H. (2010). *Acta Cryst.* E**66**, o190. 10.1107/S1600536809053707 PMC2980117 21580075

[bb4] Oxford Diffraction (2009). *CrysAlis CCD* and *CrysAlis RED* Oxford Diffraction Ltd, Yarnton, England.

[bb5] Perlovich, G. L., Tkachev, V. V., Schaper, K.-J. & Raevsky, O. A. (2006). *Acta Cryst.* E**62**, o780–o782.

[bb6] Sheldrick, G. M. (2008). *Acta Cryst.* A**64**, 112–122. 10.1107/S0108767307043930 18156677

[bb7] Shetty, M. & Gowda, B. T. (2005). *Z. Naturforsch. Teil A*, **60**, 113–120.

[bb8] Spek, A. L. (2009). *Acta Cryst.* D**65**, 148–155. 10.1107/S090744490804362X PMC2631630 19171970

